# Supplementary material for: The antifungal activity of vapour phase of odourless thymol derivate
Source: PeerJ. 2020 Aug 19;8:e9601. doi: 10.7717/peerj.9601 (PMC7443090; doi:10.7717/peerj.9601)
Supplement: Supplemental Information 1 [file peerj-08-9601-s001.pdf]

Supplementary information for:

The antifungal activity of vapour phase of odorless thymol derivate

Róbert Kubinec, [a] Jaroslav Blaško*[a], Paulína Galbavá [a], Helena Jurdáková [a], Jana Sádecká [b], Domenico Pangallo[c], Mária Bučková[c], Andrea Puškárová[c]

[a.]Faculty of Natural Sciences, Institute of Chemistry, Comenius University, Mlynská dolina CH-2, 842 15 Bratislava, Slovakia; E-Mail: jaroslav.blasko@uniba.sk
[b.]National Agricultural and Food Centre - Food Research Institute, Priemyselná 4, P.O.Box 25, 824 75 Bratislava, Slovakia.
[c.]Institute of Molecular Biology, Slovak Academy of Sciences, Dúbravská cesta 21, 845 51 Bratislava, Slovakia.
* e-mail: jaroslav.blasko@uniba.sk

Kubicin - trimethyl[5-methyl-2-(1-methylethyl)phenoxy]-silane (synonyms; TMS-thymol derivate, thymol trimethylsilyl ether); CAS#: 55012-80-1; Formula: C13H22OSi; Exact Mass: 222.1439

Table S.1 Linear retention indices I_p of thymol and kubicin measured in five different capillary columns in order of increasing stationary phase polarity.

GC column	I_p	
	**Thymol**	**Kubicin**
SPB-1	1272.6	1313.5
DB-5	1292.0	1308.3
DB-17	1487.4	1410.2
DB-23	2052.0	1510.1
DB-WAX	2202.9	1305.5

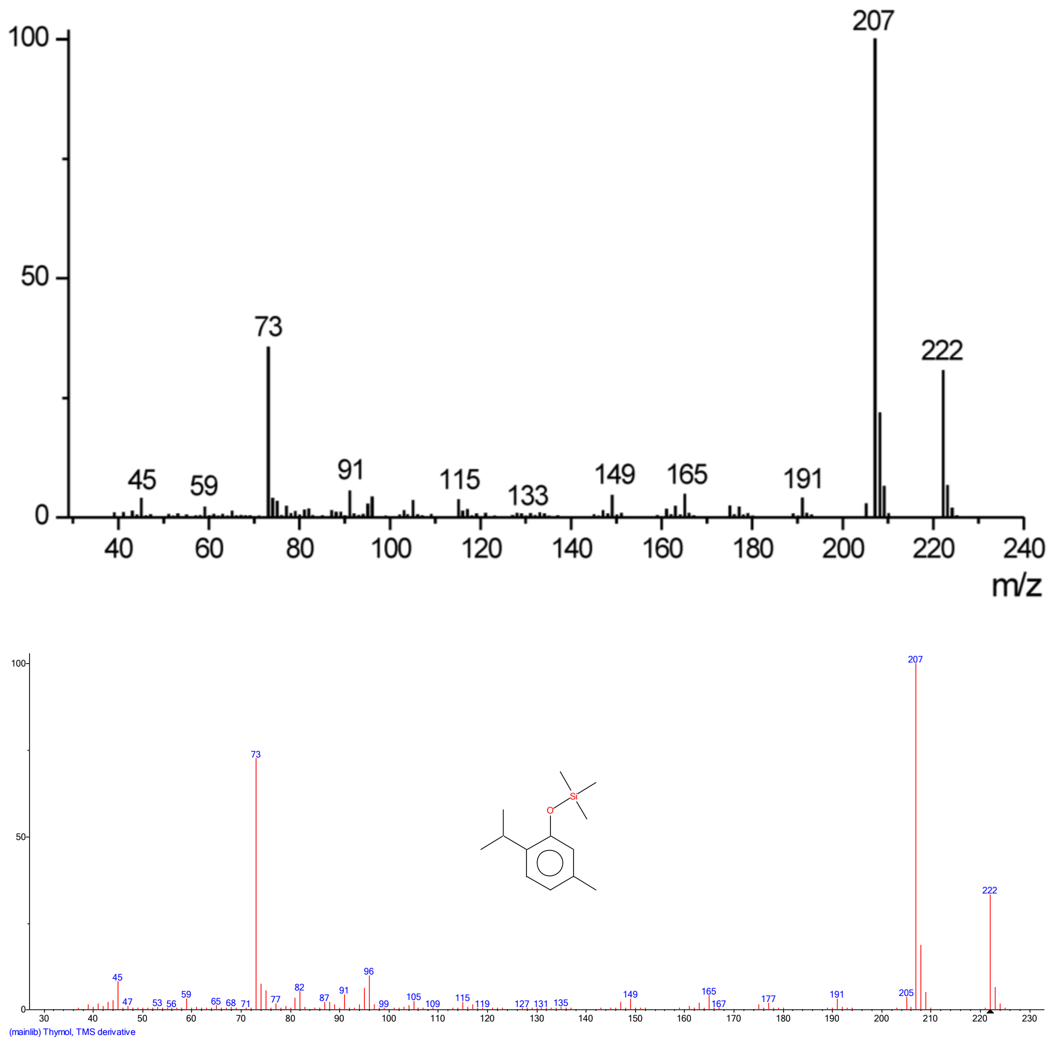

Fig. S.1 MS spectrum of kubicin (up – measured, bottom – NIST17 MS library, similarity 982)

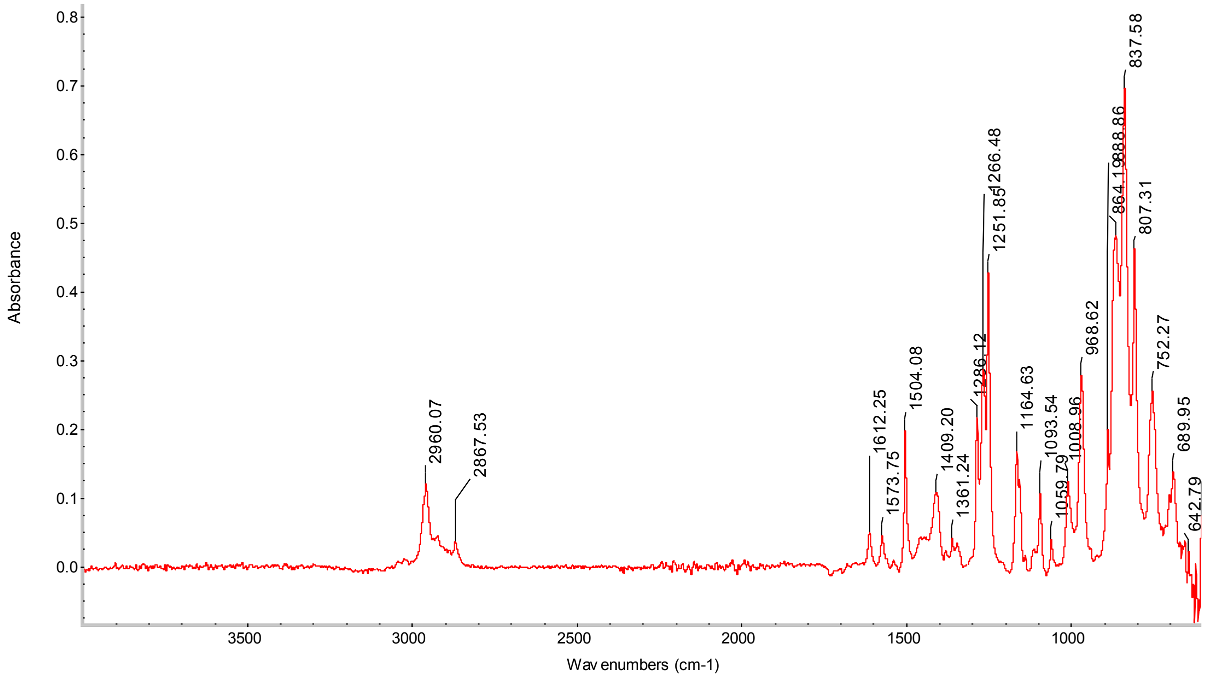

Fig. S.2 FTIR spectrum of kubicin

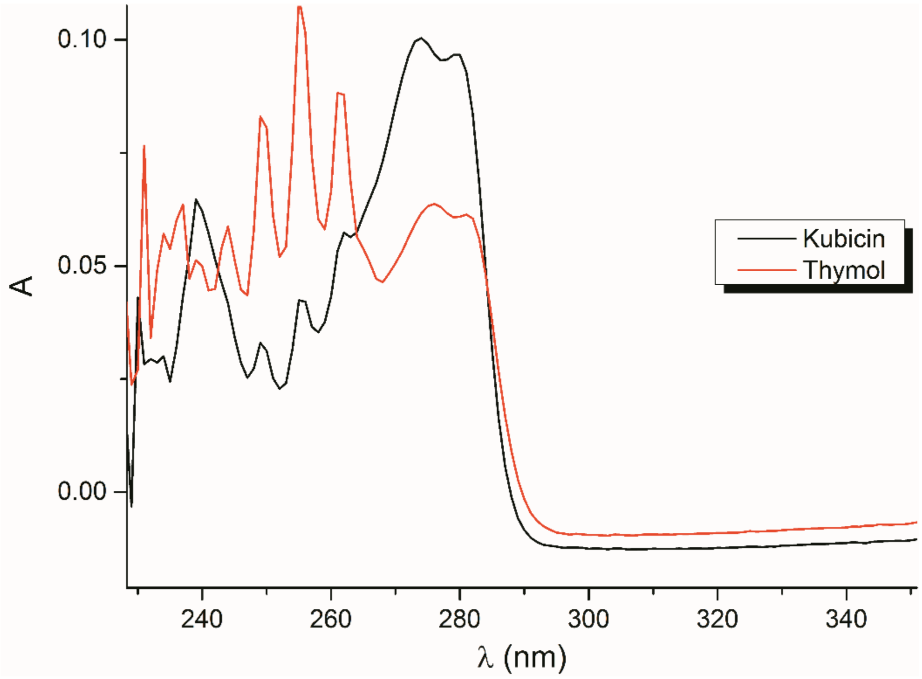

Fig. S.3 UV-VIS spectra of kubicin and thymol (10^{-4} $mol.dm^{-3}$ in chloroform)

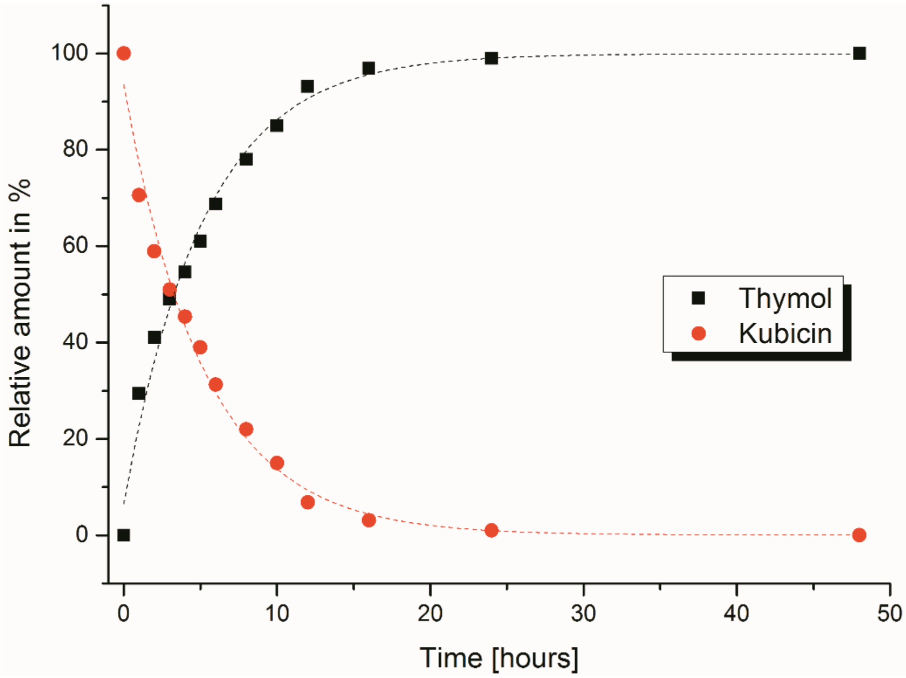

Fig. S.4 Stability time dependence of kubicin dissolved in DMSO (non-dried).

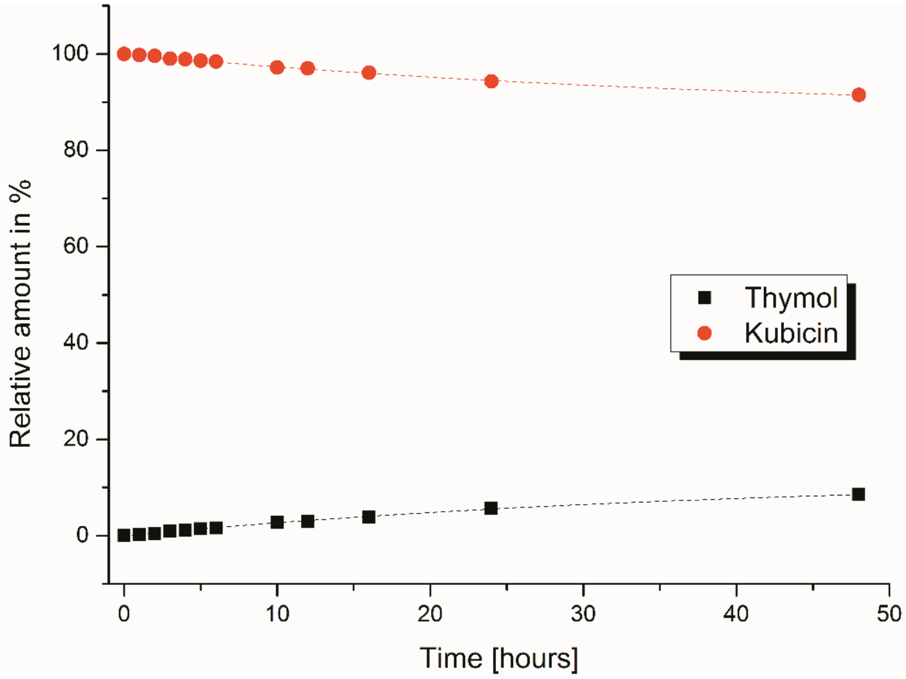

Fig. S.5 Stability time dependence of kubicin dissolved in dried DMSO.

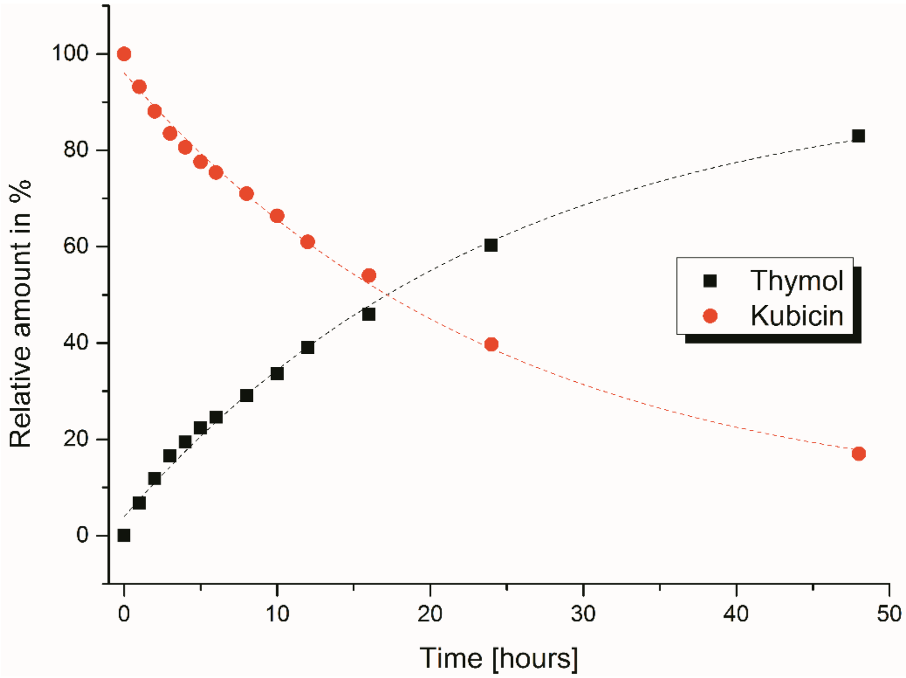

Fig. S.6 Stability time dependence of kubicin dissolved in methanol (non-dried).

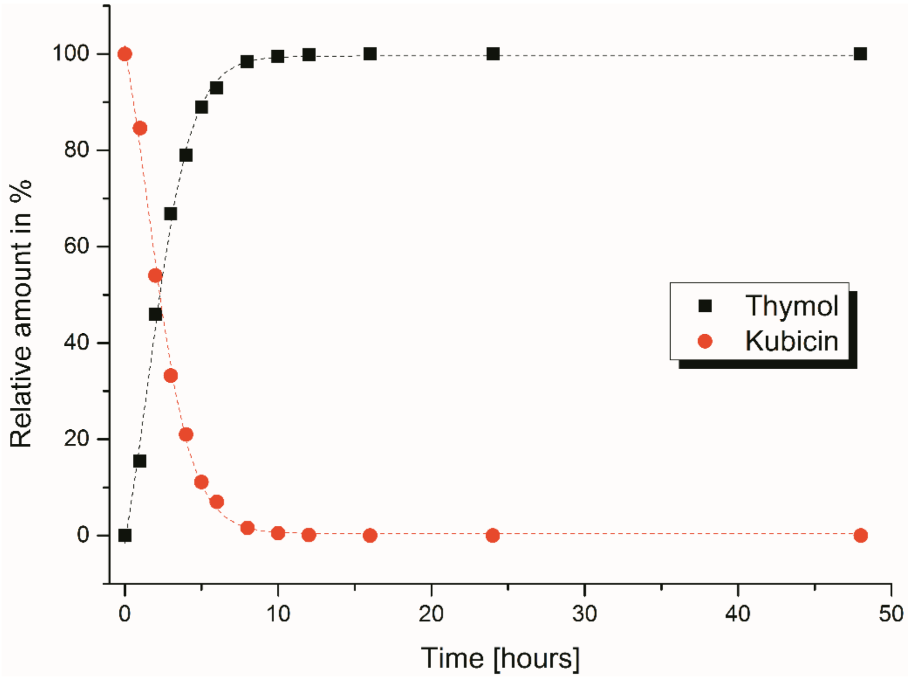

Fig. S.7 Stability time dependence of kubicin dissolved in dried methanol.

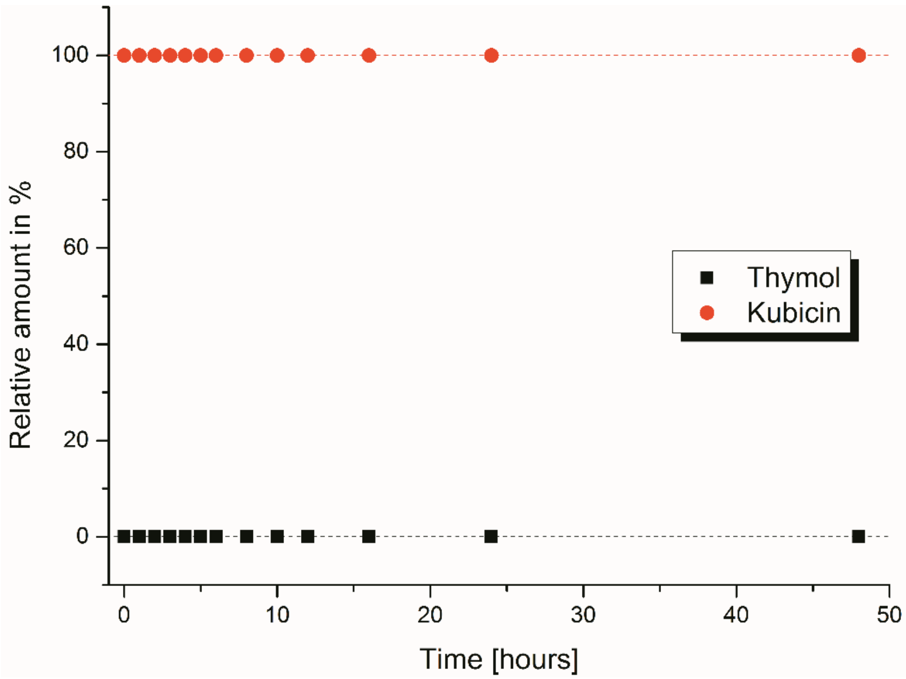

Fig. S.8 Stability time dependence of kubicin dissolved in acetone.

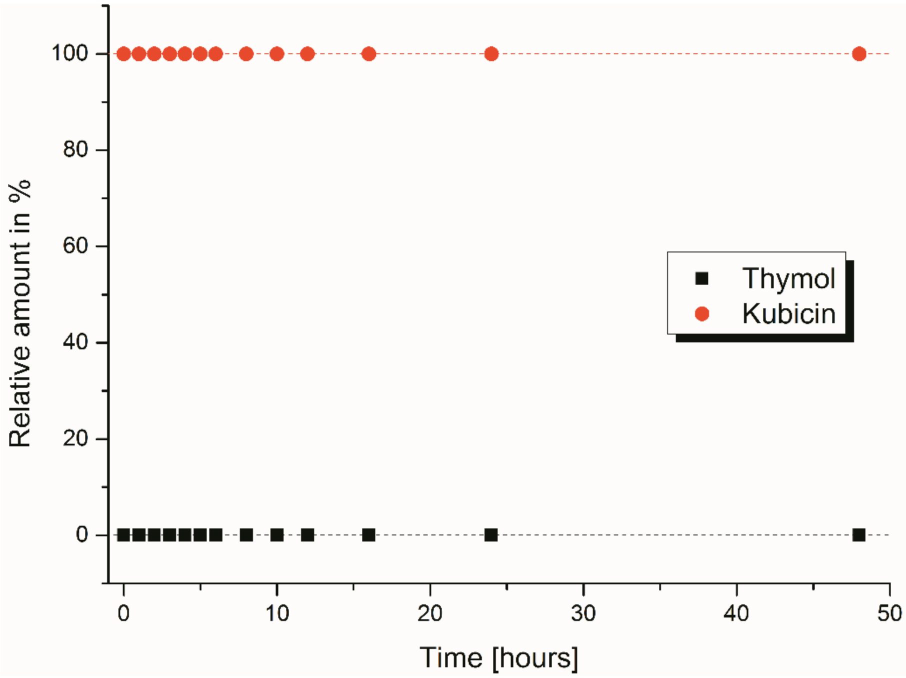

Fig. S.9 Stability time dependence of kubicin dissolved in acetonitrile.

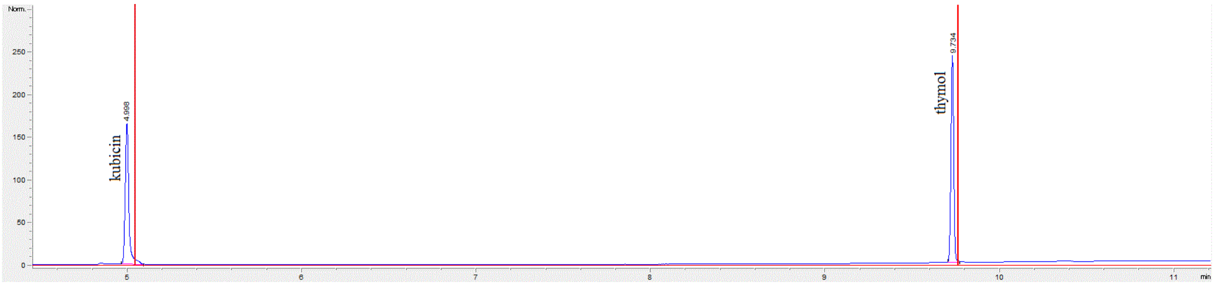

Fig. S.10 GC-O analysis for determination of odour threshold concentration of kubicin; (concentrations of kubicin and thymol in analysed mixture were 10 $mg.mL^{-1}$ acetone). FID chromatogram and aromagram (red verticals) are overlaid.
