# Supplementary material for: The antifungal activity of vapour phase of odourless thymol derivate
Source: PeerJ. 2020 Aug 19;8:e9601. doi: 10.7717/peerj.9601 (PMC7443090; doi:10.7717/peerj.9601)
Supplement: Data S2 [file peerj-08-9601-s003.pdf]

The values shown in the table were confirmed in three independent experiments in which the effect of kubicin on the growth of fungal isolates was monitored. The aim of the experiment was to determination of fungistatic and/or fungicidal effect of vapour phase of kubicin in model conditions in the presence of the test substance using these concentrations: 0.005, 0.01, 0.02, 0.04, 0.08, 0.12 μL/mL air space. The physiological effect of the test substance (kubicin) was compared to a control isolates (fungi) growing in the absence of the test substance. The same effect with the same concentration (specific for a given fungus) was observed during three different experiments.
